# Accelerated solvent extraction (ASE) for purification and extraction of silicone passive samplers used for the monitoring of organic pollutants

**DOI:** 10.1007/s11356-015-5192-1

**Published:** 2015-08-21

**Authors:** Berit Brockmeyer, Uta R. Kraus, Norbert Theobald

**Affiliations:** Federal Maritime and Hydrographic Agency (BSH), Bernhard-Nocht-Str. 78, 20359 Hamburg, Germany

**Keywords:** Silicone passive sampler, Pressurized liquid extraction, Accelerated solvent extraction, Polydimethylsiloxane, TXRF, Size exclusion chromatography (HPLC-SEC)

## Abstract

Silicone passive samplers have gained an increasing attention as single-phased, practical and robust samplers for monitoring of organic contaminants in the aquatic environment in recent years. However, analytical challenges arise in routine application during the extraction of analytes as silicone oligomers are co-extracted and interfere severely during chemical analyses (e.g. gas chromatographic techniques). In this study, we present a fast, practical pre-cleaning method for silicone passive samplers applying accelerated solvent extraction (ASE) for the removal of silicone oligomers prior to the water deployment (hexane/dichloromethane, 100 °C, 70 min). ASE was also shown to be a very fast (10 min) and efficient extraction method for non-polar contaminants (non-exposed PRC recoveries 66–101 %) sampled by the silicone membrane. For both applications, temperature, extraction time and the solvent used for ASE have been optimized. Purification of the ASE extract was carried out by silica gel and high-pressure liquid size exclusion chromatography (HPLC-SEC). The silicone oligomer content was checked by total reflection X-ray fluorescence spectroscopy (TXRF) in order to confirm the absence of the silicone oligomers prior to analysis of passive sampler extracts. The established method was applied on real silicone samplers from the North- and Baltic Sea and showed no matrix effects during analysis of organic pollutants. Internal laboratory standard recoveries were in the same range for laboratory, transport and exposed samplers (85–126 %).

## Introduction

Passive sampling, as time integrated sampling approach, is increasingly used for monitoring of organic contaminants in the water phase providing a cost-efficient alternative to active water sample collection. The basic principle of this sampling method is the passive diffusion and absorption of hydrophobic contaminants from the water phase into the sampler. A large variety of non-polar samplers have been applied so far, among those are semi-permeable membrane devices, low-density polyethylene (LDPE) strip samplers and polydimethylsiloxane (PDMS) strip samplers (Vrana et al. 2005).

Common characteristics of single-phased passive strip samplers are their simple construction, low costs and the possibility for re-use (Rusina et al. 2007). PDMS strip samplers are often the samplers of choice, because polymer-water partition coefficients (*K*_pw_) for many contaminants have been reported in the literature (Smedes et al. 2009). Furthermore, PDMS samplers can absorb chemicals with a wider log *K*_OW_ range than LDPE strips. In a current comparison study, Allan et al. (2013) tested PDMS and LDPE strips for the screening of a wide range of chemicals and showed that PDMS is less discriminating than LDPE with regard to more polar substances such as organophosphate compounds (OPCs). PDMS samplers have been successfully applied for the monitoring of polycyclic aromatic hydrocarbons (PAHs), polychlorinated biphenyls (PCBs), hexachlorobenzene (HCB) as well as OPCs in both, limnic and marine waters (Smedes 2007; Schäfer et al. 2010; Allan et al. 2013).

However, a major problem of PDMS samplers is the co-extraction of non-crosslinked silicone oligomers from the polymer. Silicone oligomers can cause considerable analytical problems like blocking of high-performance liquid chromatographic (HPLC) columns or coating of gas chromatographic (GC) liner and columns (Smedes and Booij 2012). Therefore, exhaustive pre-cleaning prior to sampling and extract purification of PDMS sampler are necessary to minimize oligomer release and subsequent interference with chemical analysis (Smedes and Booij 2012; Shahpoury and Hageman 2013; O´Connell et al. 2014). Common pre-cleaning extraction techniques, e.g. Soxhlet extraction, are solvent- and time-intensive (Schäfer et al. 2010; Smedes and Booij 2012; Shahpoury and Hageman 2013). Despite of extensive pre-cleaning steps, PDMS sampler extracts often still contain traces of non-crosslinked silicone oligomers (Smedes and Booij 2012), and hence additional purification methods, such as C-18 column chromatography or HPLC-size exclusion chromatography (SEC) are needed (Smedes and Booij 2012; Shahpoury and Hageman 2013). Thus, a more rigorous, faster extraction technique with less solvent consumption as well as an efficient PDMS extract purification method would be favourable.

In this study, accelerated solvent extraction (ASE) was used to facilitate the pre-cleaning and extraction process of silicone sheets with the aim to decrease time and solvent usage compared to the commonly used methods. Additionally, extraction and purification of PDMS sampler were optimized in order to prevent silicone coating of analytical hardware such as GC-MS. Furthermore, total reflection X-ray fluorescence (TXRF) was applied as a new, very fast and easy methodology for the detection and quantification of silicone oligomers in the final extract. The newly established methodology for passive sampler purification and extraction was applied on real marine samples.

## Materials and methods

### Experimental materials

#### Materials

Sampler strips of 55 × 90 × 0.5 mm (Altec, UK) were prepared from AlteSil silicone rubber sheets. All solvents used (acetone, acetonitrile, dichloromethane, ethylacetate, *n*-hexane, methanol and *n*-pentane) were of HPLC gradient grade or better (J.T. Baker, USA). Nitrogen 5.0 (LINDE, Germany) was used for solvent evaporation.

Surrogated and deuterated compounds (Table 1) were used as performance reference compounds (PRC), compounds that are spiked into the passive sampler prior to deployment and whose dissipation rates can be used to estimate sampling rates. Internal standards (IS) were added prior to extraction of samplers to compensate for variations during sample preparation.

**Table 1:** Internal standards (IS) and performance reference compounds (PRC) used in this study

Compound class	Standard	Compound	Abbreviation	Concentration IS (ng/mL) PRC (ng/sampler)
CHC	PRC	2,6-Dichlorobiphenyl	CB10	65.5
		Hexachlorobenzen-13C6	HCB-13C6	125.0
		2,4,6-Trichlorobiphenyl	CB30	100.0
		Lindane-13C6	HCHG-13C6	172.5
		2,2′,4,6,6′-Pentacholorbiphenyl	CB104	100.0
		2,2′,3,4,6,6′-Hexachlorbiphenyl	CB145	100.0
		1,1-Dichloro-2,2-bis-(p-chlorphenyl)ethen-d8	DDEPP-D8	100.0
		2,2′,3,4,5,5′-Hexachlorobiphenyl	CB141	100.0
		2,2′,3,4,4′,5,6,6′-Octachlorobiphenyl	CB204	80.2
	IS	ε-hexachlorocyclohexane	HCHE	5.0
		1,2,3,4-Tetrachloronaphtaline	TCN	5.0
		2,2′,3,4,5,5′,6-Heptachlorobiphenyl	CB185	5.0
PAH	PRC	Fluorene-d10	FL-D10	500.0
		Pyrene-d10	PYR-D10	500.0
		Benz[a]anthracene-d12	BAA-D12	500.0
		1,2,3-Indenopyrene-d12	I123P-D12	500.
	IS	Naphthaline-d8	NAPH-D8	40.0
		Acenaphthene-d10	ACE-D10	40.0.
		Anthracene-d10	ANT-D10	40.0
		Fluoranthene-d10	FLU-D10	40.0
		Benz[e]pyrene-D12	BEP-D12	40.0
		Benz[ghi]perylene-d12	BGHIP-D12	40.0

One sampler consists of six PDMS strips

Silica gel (Chromabond, Macherey&Nagel, Germany) was used as solid-phase material in the SPE clean-up step.

#### Instrumentation

Pressurized liquid extraction was performed with an accelerated solvent extraction system (ASE 350, Dionex, CA, USA). Ultrapure water was obtained from a Milli-Q water purification system (Integral 5 Millipore, MA, USA). Gravimetric analysis were performed on a scale with *d* = 0.001 g (Sartorius, Germany).

HPLC-SEC was performed on a system consisting of a HPLC pump (L-6200, Merck-Hitachi, Germany), an injection valve (Rheodyne), a column oven (Techlab, Germany) with a Phenogel column (5 μm, 50 A, Phenomenex, USA), a fluorescence detector (JASCO, Japan) and a fraction collector (Foxy 200 ISCO, USA).

Concentration was performed either with a parallel solvent concentrator with two backflush cooling zones (Syncore Q101, BÜCHI, Switzerland) or with nitrogen.

The silicone oligomer content was determined using a total reflection X-ray fluorescence analyzer (TXRF 3 Picotax, Röntec, Germany).

Analysis of PAHs was performed with a GC-MS system (GC CP-3800 coupled to Varian 1200, both Varian, CA, USA) using a Varian Factor Four Capillary Column VF-5 ms separation column (30-m length, 0.25-mm ID, 0.25-μm film thickness) (Varian Associates, CA, USA) in selected ion monitoring mode (SIM). Helium 5.0 (Linde, Germany) with a flow rate of 1 mL/min was used as carrier gas. The injection was performed split/splitless (split opens after 3 min), with an injection volume of 2 μL. The temperature gradient for chromatographic separation was as follows: start 60 °C for 0.2 min, temperature increase to 100 °C in 5 °C/min steps, temperature increase to 320 °C in 3.5 °C/min steps and a final constant temperature for 4 min. The injection was carried out with a cold injection system with a starting temperature of 60 °C, following an increase of 10 °C/s to a final temperature of 280 °C. The temperatures of transfer line, ion source and quadrupole were 275, 250, and 40 °C, respectively. The quantification of target analytes was performed with internal standards.

Determination of chlorinated hydrocarbons (CHCs) was performed with a GC-MS ion-trap system (GC CP3800 coupled to a Saturn 2200, both Varian, CA, USA) in the multi-reaction monitoring mode (MRM). As separation column, a HT 8 (0.22 mm ID, 25 m length, 0.22 μm film thickness) (SGE Analytical Science, Milton Keynes, UK) was used, with a desactivated pre-column (2- to 5-m length, 0.53-mm ID) (Agilent, Germany).

Helium 5.0 (Linde, Germany) with a flow rate of 1.3 mL/min was used as carrier gas. The injection was performed split/splitless (split opens after 3 min), with an injection volume of 2 μL. The temperature gradient for chromatographic separation was as follows: start 90 °C for 1 min, temperature increase to 170 °C in 10 °C/min steps, temperature increase to 290 °C in 3 °C/min steps and a final constant temperature for 40 min. The injection was carried out with a cold injection system with a starting temperature of 90 °C, following an increase of 5 °C/s to a final temperature of 250 °C. The temperatures of trap, manifold and transfer line were 200, 80, and 280 °C, respectively. The axiale modulation of the ion-trap was 4.1 V. The quantification of target analytes was performed with internal standards.

### Optimizing ASE parameters for silicone rubber pre-cleaning

ASE is used for the first time as a pre-cleaning tool for PDMS strips in this study. The release of small polymers from silicone rubber (oligomers) by different solvents was tested for eight solvents and combinations, which are used typically for pre-cleaning (ethylacetate, acetone, *n*-hexane, methanol/*n*-pentane (1:1), acetonitrile/methanol (2:1), dichloromethane/acetone (1:1), *n*-hexane/acetone (1:1 *v*/*v*) and *n*-hexane/acetone (3:1 *v*/*v*)) (Rusina et al. 2007; Schäfer et al. 2010; Ezzell and Richter 2012; Smedes and Booij 2012; Allan et al. 2013). Past studies investigated polymer swelling in organic solvents, which lead to an enhanced fragility of PDMS strips and blockage of solvent flow through the ASE extraction cell (Rusina et al. 2007; Shahpoury and Hageman 2013). Thus, oligomer release and swelling was quantified by gravimetric analysis. Pre-weighted silicone strips (55 × 90 × 0.5 mm) were folded into extraction cells (22 mL) with glass fibre filters in the screw cap and extracted with each solvent (−mixture) at 100 °C using the parameters given in Table 2.

**Table 2:** ASE instrumentation parameters for different experimental approaches

	Temperature (°C)	Static time (min)	Cycles
Solvent tests	100	10	2
Time series	100	10–90 (10-min steps)	1
Temperature tests	75; 100; 125	10	5

All extractions were performed with 1500 psi pressure, 50 % flush and 5 min oven heat

After extraction, strips were wiped with a paper tissue (Kimtech, Kimberly-Clark), quickly wrapped in pre-weighted aluminium foil to prevent evaporation of absorbed solvent and weighted. Gravimetric results were used to quantify swelling as the volume increases after extraction relatively to the initial polymer volume, taking the loss of oligomers into account. Finally, strips were air dried in a fume hood to evaporate absorbed solvent and re-weighted. The mass of released oligomers was calculated from the initial mass of the strip and the mass after extraction and drying.

In addition, time and temperature of extraction were optimized to achieve the highest release rates of oligomers. Therefore, (i) time series extraction experiments (*n* = 3) where the static extraction step increased in 10 min steps from 10 up to 90 min, at constant temperature (100 °C) and (ii) extraction experiments at different temperatures (75, 100 and 125 °C) but constant extraction time (50 min) were performed with *n*-hexane/acetone (1:1 *v*/*v*).

For a reliable evaluation of the clean-up efficiency with ASE, a comparison was made with classical clean-up procedures, e.g. Soxhlet extraction or extraction by shaking (Table 3). For comparable results, all clean-up methods were performed two times with three strips each cut from the same PDMS sheet.

**Table 3:** Comparison of different silicone rubber pre-clean-up methods regarding solvent and time

Clean-up method	According to reference	Total solvent (mL)	Time (h)	Swelling (%)	Release of oligomers (%)
Soxhlet extraction ethylacetate	Smedes and Booij (2012)	400	100	92	2.5
Extraction ethylacetate	Shahpoury and Hageman (2013)	400	48	76	2.4
Extraction *n*-hexane/acetone (3:1)	Schäfer et al.(2010)	800	96	124	2.5
ASE *n*-hexane/acetone (1:1)	This study	169	1.2	42	2.2

Results of experimental approach using different clean-up methods in regard to swelling and the associated silicone oligomer release in %

### Sample extraction

**Fig. 1:**
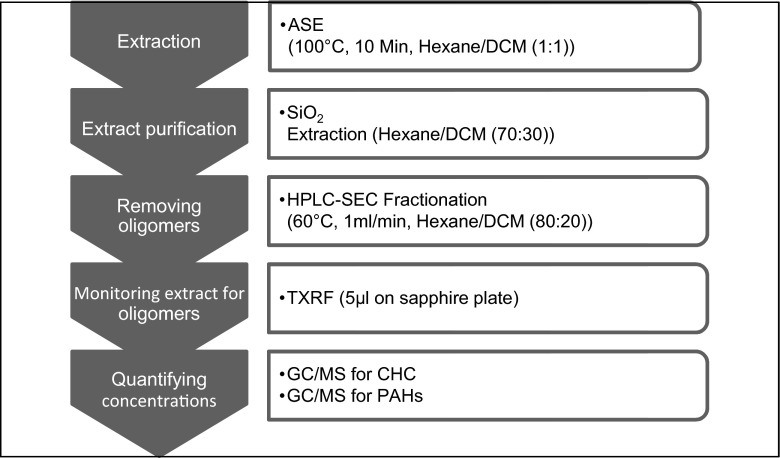
Scheme of silicone rubber sample extraction, clean-up and analysis

The main aim during extraction and purification of PDMS samplers is to achieve good recovery rates of analytes, as well as to completely remove non-crosslinked silicone oligomers. In order to gain optimized recovery rates of CHCs and PAHs, the same organic solvent should be used for the entire extraction and clean-up process (Fig. 1) to avoid target compound losses due to solvent exchange during the extraction process. In contrast to the pre-cleaning step (s.a.), where the solvent should have a high oligomer release capacity, organic solvent for sampler extraction should yield a minimum oligomer release.

Therefore, in a first step, different solvents (*n*-hexane/acetone (1:1 *v*/*v*), dichloromethane, acetonitrile and acetonitrile/methanol (2:1 *v*/*v*)) typically used for non-polar contaminants extraction from passive sampler (Rusina 2009; Schäfer et al. 2010; Smedes and Booij 2012; Shahpoury and Hageman 2013) were tested twice for their extraction efficiency for analytical target compounds by using ASE. Briefly, ASE cells were filled with sea sand as filling matrix and spiked with IS. Each sample was extracted in 3 cycles of 5-min static time (100 °C) to find the optimal extraction time for a complete extraction of analytes. An azeotropic solvent exchange from the more polar solvents acetonitrile and methanol to hexane was performed with an excess of hexane according to Smedes and Booij (2012).

In a second step, extraction tests were performed on pre-cleaned PDMS sheets spiked with PRCs (Table 1) in a methanol/water mixture (90:10 *v*/*v*) according to Rusina (2009) and shaken for 14 days, while the water content was increased to methanol/water (1:1 *v*/*v*) after 1 week. Spiked PDMS sheets were ASE extracted, whereas each ASE cell (100 mL) was filled with six spiked PDMS sheets (1 sample), filled up with pre-combusted sea sand (Merck, Darmstadt, Germany) and IS. Extraction was performed with optimized solvent, temperature and time (1 × 10 min, 100 °C, hexane/dichloromethane (1:1 *v*/*v*)). These PDMS sheets represent the fabrication blank with no further transportation or deployment in water.

In a third step, the newly developed method (Fig. 1) was applied on field samples which have been deployed in marine waters of the German Bight (Heligoland waters) for 43 days and in brackish waters of the Baltic Sea (Fehmarn waters) for 63 days. Field samples comprised each of a set of two deployed sampler and a transport blank and enabled the direct comparison of real sampler matrix with laboratory sampler blanks.

All extracts were evaporated to 1 ml by parallel solvent reduction and further purified (“Extract purification and analysis”) prior to GC-MS analysis.

### Extract purification and analysis

The ASE sample extracts need additional purification steps to remove last traces of silicone oligomers and co-extracted material (e.g. organic matter) from field samples. Purification from co-extracted material was performed by SPE using 500-mg silica gel. Target compounds were eluted with hexane/dichloromethane (70:30, 5 mL) and evaporated to 1 ml by a gentle stream of nitrogen. Extracts were further purified by HPLC-SEC. Co-extracted silicone oligomers were separated by HPLC-SEC (injection volume 0.5 mL) with hexane/dichloromethane (80:20), whereby the first fraction (0–10 min) contained the non-crosslinked silicone oligomers and the second fraction the CHCs and PAHs fraction (10–28 min). Column temperature was set to 60 °C to avoid adhesion of silicone in the system. The second fraction was concentrated to 0.2 mL with nitrogen and monitored for its oligomer load with TXRF before instrumental analysis. For this, an aliquot of 5 μL was slowly dropped onto pre-cleaned sapphire disc, allowing the organic solvent time to evaporate. The disc was loaded into the TXRF and measured for 1000 s for its silicium load, which is the representative element of the silicone oligomer. A rubidium reference standard was used for instrument calibration. If the silicium load of the sample was in the range of SEC blanks, an analysis of CHCs (GC-MS/MS) and PAHs (GC-MS) was performed. The complete extraction and analysis process is illustrated in Fig. 1.

## Results and discussion

### Optimization of the silicon rubber pre-cleaning

**Fig. 2:**
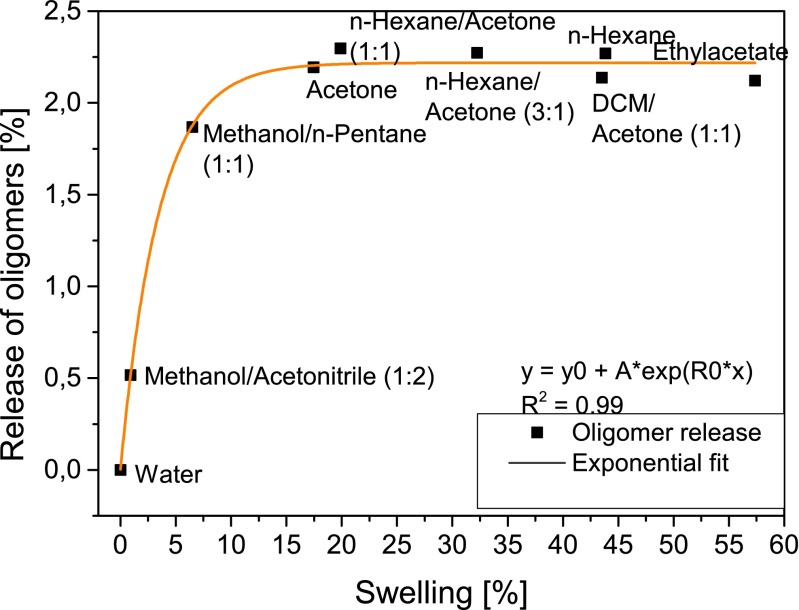
Release of oligomers as a function of swelling for different organic solvents (water, methanol/acetonitrile, methanol/pentane, acetone, hexane, hexane/acetone, dichloromethane/acetone, ethylacetate) using ASE (100 °C, 2 × 10 min)

Different solvents showed different patterns of swelling and of total oligomer release (Fig. 2). No swelling and release of oligomers was induced by water, while up to 2.3 % of silicone oligomers were released when non-polar solvents were used (*n*-hexane/acetone (1:1 *v*/*v*)) (Fig. 2). The release of oligomers was exponentially dependent on the swelling of the silicone strips (*R*^2^ = 0.99) (Fig. 2), from 0 to 60 %. In contrast to a study of Shahpoury and Hageman (2013), none of the used solvents expanded the silicone rubber as drastically as to block the solvent flow. However, the blockage might depend on the volume of silicone rubber relative to the volume of the extraction cell. Rusina et al. (2007) showed that PDMS strips became more breakable after increased swelling. Thus, the 1:1 combination of hexane/acetone was used as the solvent of choice in further pre-cleaning experiments, providing a high oligomer release rate with medium swelling (20 %). In all experiments, PDMS strips regained their original size and strength after the drying process (evaporation of the solvent).

**Fig. 3:**
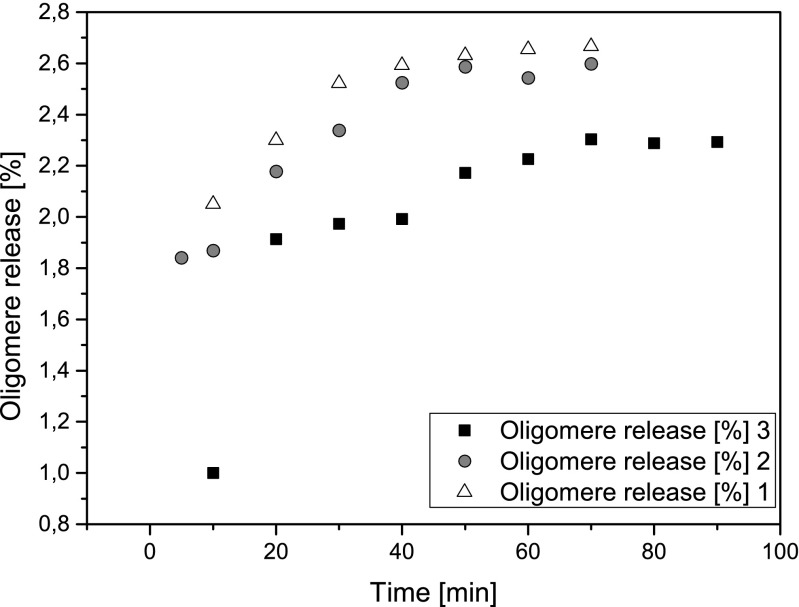
Time-dependent release of silicone oligomers from silicone strips of different batches using ASE at constant temperature and solvent (100 °C, hexane/acetone (1:1))

It was found that the amount of released oligomers from silicone rubber strips increased with increasing extraction time up to 70 min with no further release with increasing time (Fig. 3), indicating the oligomer release from the silicone rubber strips to be fairly exhausted. The final amount released is dependent on the batch from which the passive sampler strips were prepared.

The variation of temperature showed no differences in the amount of released oligomers and hence was excluded as a factor for optimization of oligomer release rates. Thus, the ASE default temperature of 100 °C was applied.

Additionally to the determination of the weight of the PDMS strips, corresponding extracts were analysed by GC-MS to proof the differences in weight to be a result of oligomer release. All extracts of silicone rubber pre-cleaning showed the typical repeating peaks of silicone oligomers, with the peaks containing the ions m/z 73, 147, 221, 282, 355 and 429, finally leading to silicone coating of GC liner and column.

In all different pre-clean-up procedures used, a significant amount of mass (>2 %) was released from the PDMS strips (Table 3). While the swelling of the PDMS strips was highly variable from 42 % (ASE) to 124 % (extraction with hexane/acetone), release rates were quite uniform, all above 2 %, ranging from 2.2 % (ASE) to 2.5 % (Soxhlet extraction with ethylacetate and extraction with hexane/acetone).

Silicone rubber pre-cleaning with ASE takes only 70 min and is thus much faster as other classical pre-cleaning methods like Soxhlet or shaking extraction (36–100 h) (Smedes and Booij 2012; Shahpoury and Hageman 2013). Thus, ASE-based methods enhance the suitability of silicone rubber samplers for routine applications profoundly as it reduce labour time considerably. In addition, no long-term preparation of campaigns is necessary, making this sampler type suitable for deployments on short notice (which is not possible using traditional clean-up methods).

Although the silicone sheets have been thoroughly pre-cleaned, traces of oligomers are still co-extracted during sampler extraction after deployment. Quantitative determination of silicones was not carried out by GC or GC-MS because they quickly destroy the GC performance. Instead, we found that TXRF proved to be a very fast and easy procedure to detect and quantify silicone oligomers. Measurements with TXRF revealed that more than 90 % of extractable silicone oligomers could be removed by the pre-cleaning step with ASE similar to other cleaning procedures. Thus, further cleanup of the sample extract is still mandatory to avoid instrument interferences, such as silicone coating of gas chromatography (GC) liners.

### Optimization of the organic solvent for ASE extraction

**Fig. 4:**
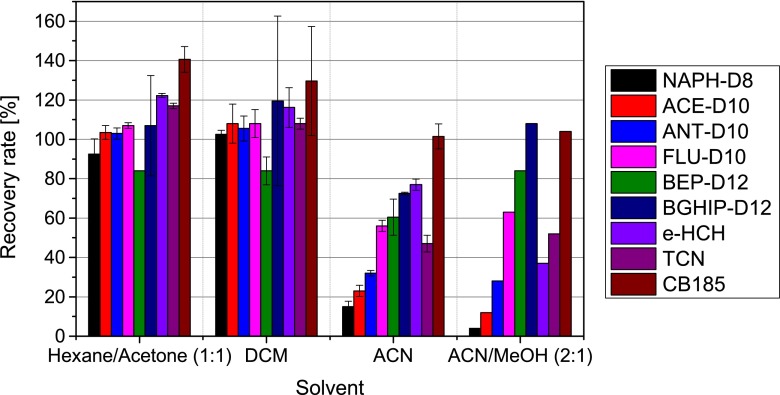
Recovery rate of internal CHC and PAH standards using different solvents (dichloromethane, acetonitrile) and solvent mixtures (*n*-hexane/acetone (1:1 *v*/*v*); acetonitrile/methanol (2:1 *v*/*v*)) by ASE extraction (*n* = 2 for each solvent (−mixture))

Results of the solvent extraction experiments using ASE showed that the analytes were extracted within the first 10 min. The recovery rates of IS (CHC and PAH) increased with decreasing polarity of the solvent (Fig. 4). Within the more polar solvents (acetonitrile and acetonitrile/methanol) recovery rates were better for aromatic compounds with 4- or more rings like fluoranthene, benz[e]pyrene and benz[ghi]perylene (56–108 %) than for the more volatile 2- and 3-ring aromatic compounds naphthalene, acenapthene and anthracene (4–32 %).

Polar solvents need to be transferred to non-polar solvents as recommended by Smedes and Booij (2012), and this additional step may be responsible for lower recovery yields of lower boiling point analytes (2- and 3-ring PAH, ε-HCH and TCN). Thus, in terms of better extract efficiencies and higher recoveries of non-polar contaminants, non-polar extraction solvents such as hexane or dichloromethane should be used. However, non-polar extraction solvents co-extract more silicone oligomers, simultaneously. Therefore, an additional cleanup step is necessary, which was performed by HPLC-SEC as described in “Removing silicone oligomers from sample extracts by HPLC-SEC”.

### Removing silicone oligomers from sample extracts by HPLC-SEC

Measurements of PDMS extracts with TXRF showed co-extraction of silicone oligomers (see “Optimization of the silicon rubber pre-cleaning”). Hence, extracts had to be further purified to avoid coating of instrumental parts. Smedes and Booij (2012) recommend extract purification with C-18 bonded silica cartridges, which is suitable for methanol or acetonitrile/methanol as solvent. Due to the fact that the ideal solvent for PDMS sampler extraction with ASE was found to be non-polar (“Optimization of the organic solvent for ASE extraction”), alternative purification steps needed to be performed. Shahpoury and Hageman (2013) used SEC to remove silicone oligomers from sample extracts only for PAH analysis. HPLC-SEC can be set up with non-polar solvents and hence no solvent transfer from non-polar to polar and back is necessary, which can potentially result in lower analyte recoveries by using C-18 silica cartridges. In this study, HPLC-SEC was tested for the separation of PAH and CHCs from silicone oligomers using a mixture of hexane-dichloromethane as eluent in order to keep the same extraction solvents during the entire extraction procedure. Monitoring the resulting SEC fractions with TXRF showed that the majority of silicone oligomers (mean 98.9 %) were present in the first fraction (0–10 min). Less than 1 % (average 0.3 %) of the original silicone oligomer content was left in the second fraction (10–28 min), in which the target CHCs and PAHs were eluted. HPLC-SEC is restricted to a certain amount of residual oligomers, e.g. if the pre-cleaning step to remove residual oligomers is skipped, the burden for a semi-preparative column to separate target compounds from oligomers is too high. HPLC-SEC thus proved to be a very efficient purification method to remove co-extracted silicone oligomers showing the additional advantage that no solvent exchange is necessary.

Additionally to HPLC-SEC, the fast monitoring of the silicone content in each (purified) extract by TXRF avoids analytical interferences and coating of instrument parts (e.g. GC liner and column).

### Field samples

#### Recovery rates

**Fig. 5:**
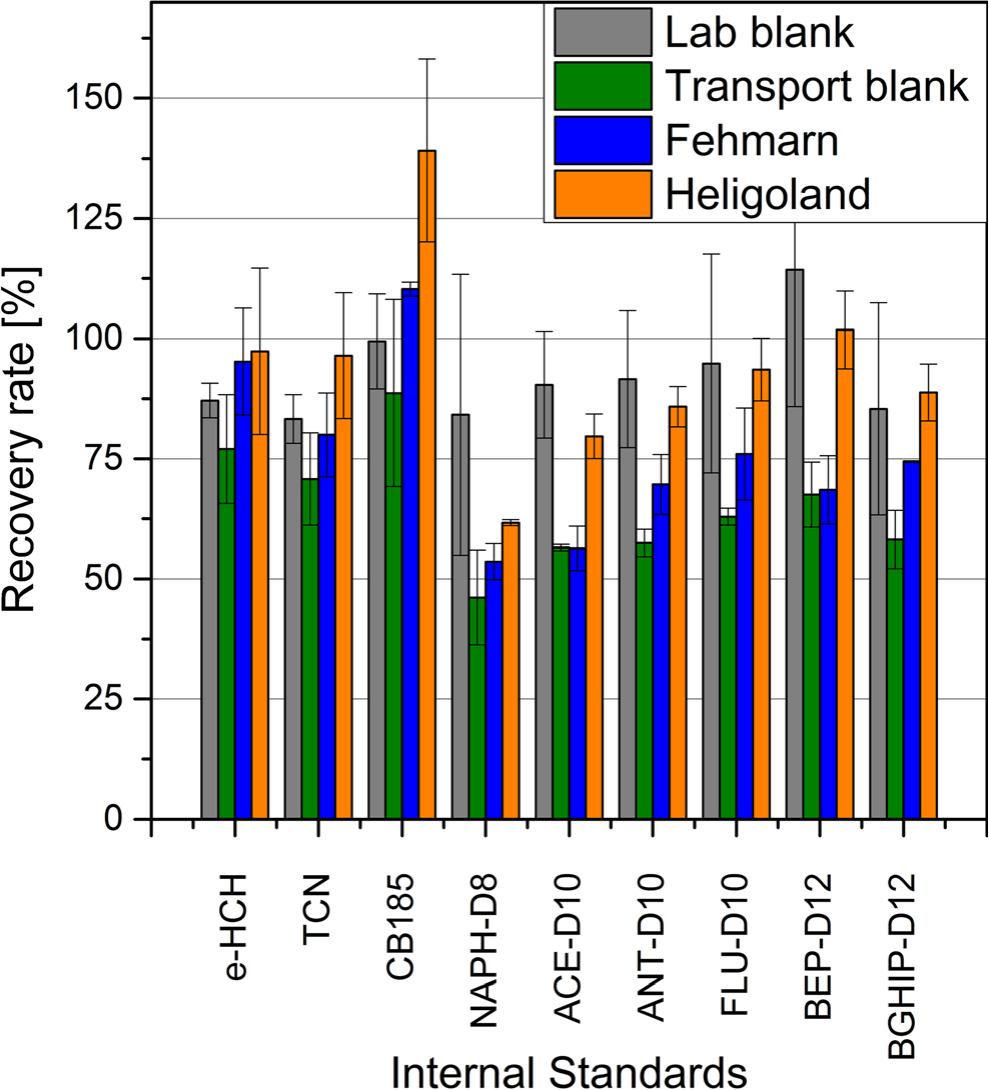
Recovery rates of internal laboratory standards on deployed samplers from Fehmarn and Heligoland (*n* = 2 each) and their corresponding lab (*n* = 3) and transport blanks (*n* = 2)

The whole set of deployed sampler, transport and laboratory blanks was extracted and purified as described in “Sample extraction”. Internal laboratory standard recoveries represent the quality of the extraction and purification procedure and ranged from 83–114 % for CHCs and PAHs (Fig. 5). Deployed PDMS samplers from Heligoland and Fehmarn also showed good IS recovery rates for CHCs in the range from 85 to 126 % (Fig. 5), which is within the range of the corresponding lab and transport blanks. In general, PAH recovery was good, while 2- and 3-ring aromatic PAHs showed lower recoveries than PAHs with higher aromatic rings.

The recovery rates of PRCs in laboratory blanks can be additionally used for QA/QC issues, for example, to ensure a successful spiking procedure and storage. The recovery rates of PRCs determined in laboratory blanks ranged from 66 to 101 % demonstrating that the used spiking procedure was successful for most of the CHCs and PAHs. Due to its physico-chemical properties, γ-HCH-^13^C_6_ showed the lowest recovery as this analyte might be already equilibrated with the water phase during spiking, supported by the fact that the corresponding internal standard ε-HCH, representing the extraction efficiency, showed good recoveries (87 %).

Overall, the results of the recovery rates proofed that matrix of deployed samplers does not affect extraction and purification as well as that the used procedure is reliable for PDMS extraction, avoiding the interference of silicone oligomers during mass spectrometric analysis. Full-scan MS measurements revealed no additional background peaks which could be related to silicone oligomers.

#### CHC and PAH concentration

**Fig. 6:**
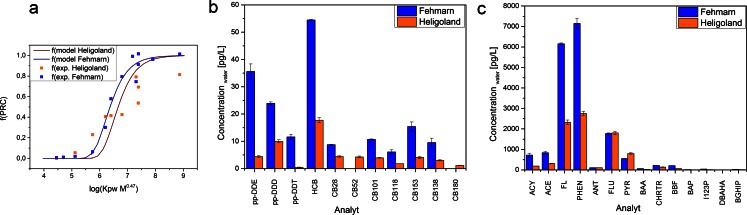
**a** Fits of the proportionality constant *B* by nonlinear least square regression. **b**, **c** Calculated concentrations of CHCs and PAHs in water from deployed sampler (*n* = 2) of Fehmarn (Baltic Sea) and Heligoland (North Sea)

At the stations Fehmarn and Heligoland CHC and PAH concentrations in the water phase were estimated according to the method of Smedes and Booij (2012). Briefly, the proportionality constant *B* was calculated by a nonlinear least square regression calculated by the PRC fractions retained in the exposed sampler compared with the control sampler versus log(*K*_PW_*M*^0.47^) as presented in Fig. 6a. The proportionality constant *B* was then used to estimate the specific sampling rate which is necessary to calculate the water phase concentrations of the target compounds.

Heligoland represents a marine station while Fehmarn represents a brackish water station. Thus, as expected, Fehmarn waters generally show higher analyte concentrations than Heligoland waters (Fig. 6b, c). The concentrations of individual CHCs were between 6–54 and 0.4–18 pg/L for Fehmarn and Heligoland, respectively, with hexachlorobenzene having the highest concentration. The concentrations of individual PAHs ranged from 3–7145 and 1–2750 pg/L for Fehmarn and Heligoland, respectively. Phenanthrene, fluorene and fluoranthene have the highest concentration within the measured PAH compounds. The more hydrophobic PAHs (e.g. indenopyrene) have minor concentrations. In general, the concentrations are in the same range and show the same compound patterns and proportions compared to active water samples of routine monitoring operated by the BSH (Loewe et al. 2013; MURSYS 2011). Hence, PDMS sampler deployment and extraction as described in this study is suitable for routine monitoring of non-polar organic contaminants in brackish and seawater.

## Conclusions

The method presented improves handling and use of silicone rubber sheets for passive sampling in several aspects: (1) ASE for pre-cleaning and extraction, (2) HPLC-SEC for the removal of residual silicone oligomers and (3) control of oligomers by TXRF.

ASE can perform pre-cleaning and extraction in a fraction of time and with much less solvent compared to other extraction procedures, such as Soxhlet. Furthermore, non-polar extraction solvents show better extraction efficiencies and higher analyte recoveries than polar solvents. In combination with HPLC-SEC as additional sample extract purification procedure, residual silicone oligomers can be removed before the determination of non-polar compounds. By monitoring the silica content in the extract with TXRF, interferences of chemical analysis can be avoided. The entire method was successfully applied on deployed marine sampler. In conclusion, this study optimized the preparation as well as extraction and purification procedure of PDMS samplers in order to enable silicone passive samplers more favourable and robust for routine monitoring of contaminants in the water phase.

## References

[CR1] Allan IJ, Harman C, Ranneklev SB, Thomas KV, Grung M (2013). Passive sampling for target and nontarget analyses of moderately polar and nonpolar substances in water. Environ Tox Chem.

[CR2] Ezzell J, Richter B (2012). Extraction of PCBs from environmental samples using accelerated solvent extraction. Application note, Thermo Fisher Scientific.

[CR3] Loewe P, Klein H, Weigelt-Krenz S (2013). System North Sea–2006 & 2007: status and trends. Berichte des BSH 49, Bundesamt für Seeschifffahrt und Hydrographie.

[CR4] MURSYS (2011) Regional distribution of organic contaminants in the pelagial and the surface sediment of the Baltic in 2011. Marine environment reporting system Germany., http://www.bsh.de/Vorlagen/ressources/Druckversion.jsp?_PRINTPAGE_=yes&_PRINTOID_=146351 **& Accessed 16 March 2015**

[CR5] O´Connell SG, McCartney MA, Paulik LB, Allan SE, Tidwell LG, Wilson G, Anderson KA (2014). Improvements in pollutant monitoring: optimizing silicone for co-deployment with polyethylene passive sampling devices. Environ Poll.

[CR6] Rusina TP (2009). New methods of sampling and sample pre-concentration of hydrophobic compounds in aquatic ecosystems. Magnifying passive sampling aspects.

[CR7] Rusina TP, Smedes F, Klanova J, Booij K, Holoubek I (2007). Polymer selection for passive sampling: a comparison of critical properties. Chemosphere.

[CR8] Schäfer RB, Hearn L, Kefford BJ, Mueller JF, Nugegoda D (2010). Using silicone passive samplers to detect polycyclic aromatic hydrocarbons from wildfires in streams and potential acute effects for invertebrate communities. Water Res.

[CR9] Shahpoury P, Hageman KJ (2013). Pressurized liquid extraction of polycyclic aromatic hydrocarbons from silicone rubber passive samplers. J of Chromatogr A.

[CR10] Smedes F, Greenwood R, Mills G, Vrana B (2007). Monitoring of chlorinated biphenyls and polycyclic aromatic hydrocarbons by passive sampling in concert with deployed mussels. Compre. Anal. Chem. 48.

[CR11] Smedes F, Booij K (2012). Guidelines for passive sampling of hydrophobic contaminants in water using silicone rubber samplers. ICES Techniques in Marine Environ Sci.

[CR12] Smedes F, Geertsma RW, Tvd Z, Booij K (2009). Polymer-water partition coefficients of hydrophobic compounds for passive sampling: application of cosolvent models for validation. Environ Sci & Technol.

[CR13] Vrana B, Allan IJ, Greenwood R, Mills GA, Dominiak E, Svensson K, Knutsson J, Morrison G (2005). Passive sampling techniques for monitoring pollutants in water. Trends in Anal. Chem..

